# Hyper-hippocampal glycogen induced by glycogen loading with exhaustive exercise

**DOI:** 10.1038/s41598-018-19445-4

**Published:** 2018-01-19

**Authors:** Mariko Soya, Takashi Matsui, Takeru Shima, Subrina Jesmin, Naomi Omi, Hideaki Soya

**Affiliations:** 1 Laboratory of Exercise Biochemistry and Neuroendocrinology, Faculty of Health and Sport Sciences, University of Tsukuba, Tsukuba 305-8574, Ibaraki, Japan; 2 Department of Sport Neuroscience, Advanced Research Initiative for Human High Performance (ARIHHP), Faculty of Health and Sport Sciences, University of Tsukuba, Tsukuba 305-8574, Ibaraki, Japan; 3 Laboratory of Exercise Nutrition, Faculty of Health and Sport Sciences, University of Tsukuba, Tsukuba 305-8574, Ibaraki, Japan; 4 Department of Body, ARIHHP, Faculty of Health and Sport Sciences, University of Tsukuba, Tsukuba 305-8574, Ibaraki, Japan

## Abstract

Glycogen loading (GL), a well-known type of sports conditioning, in combination with exercise and a high carbohydrate diet (HCD) for 1 week enhances individual endurance capacity through muscle glycogen supercompensation. This exercise-diet combination is necessary for successful GL. Glycogen in the brain contributes to hippocampus-related memory functions and endurance capacity. Although the effect of HCD on the brain remains unknown, brain supercompensation occurs following exhaustive exercise (EE), a component of GL. We thus employed a rat model of GL and examined whether GL increases glycogen levels in the brain as well as in muscle, and found that GL increased glycogen levels in the hippocampus and hypothalamus, as well as in muscle. We further explored the essential components of GL (exercise and/or diet conditions) to establish a minimal model of GL focusing on the brain. Exercise, rather than a HCD, was found to be crucial for GL-induced hyper-glycogen in muscle, the hippocampus and the hypothalamus. Moreover, EE was essential for hyper-glycogen only in the hippocampus even without HCD. Here we propose the EE component of GL without HCD as a condition that enhances brain glycogen stores especially in the hippocampus, implicating a physiological strategy to enhance hippocampal functions.

## Introduction

Glycogen is an important energy source for muscle during exercise, and it is depleted with increased intensity and/or duration of exercise^1^. Such glycogen depletion leads to muscle fatigue during endurance exercise^2–4^. To avoid muscle fatigue, muscle glycogen-loading (GL) – a well-established sports conditioning strategy including both exercise and diet for 1 week before competition – increases muscle glycogen levels and enhances the endurance capacity in humans and animals^5–8^.

GL has been developed using a popular theory called “muscle glycogen supercompensation”, which is characterized by an initial depletion of muscle glycogen levels followed by a considerable replenishment of muscle glycogen 24–48 hours after acute exercise^9–11^. Åstrand first proposed the classic GL protocol: 3 days of exercising and a low-carbohydrate diet to induce muscle glycogen depletion followed by 3 days of a high-carbohydrate diet for hyper muscle glycogen^12^. However, classic GL protocol is complicated and occasionally induces restlessness with hypoglycemia due to the 3 days of low carbohydrate diet^5,13^. To solve these problems, Sherman *et al*. established a novel GL protocol using only a high-carbohydrate diet and exercises that induced hyper muscle glycogen mirroring the classic protocol^7^. This novel GL protocol is popular among modern endurance athletes^14,15^.

Interestingly, similar type of glycogen supercompensation phenomena in the brain were observed in our recent study^16^. We found that brain glycogen decreases with exhaustive exercise^17^, particularly in the hippocampus and cortex, and that supercompensation occurs, as it does in muscle, 6 hours after exhaustive exercise in rats^16^. These elevated glycogen levels were sustained for up to 24 hours after exhaustive exercise^16^. Therefore, we have hypothesized that GL increases brain glycogen storage, as observed in muscle, based on the exercise-induced glycogen-supercompensation theory as mentioned above.

Brain glycogen, which is localized in astrocytes and produces lactate as a neuronal energy source and/or neuromodulator, plays a critical role in memory function and exercise endurance^18–22^. Chronic exercise that enhances endurance capacity and cognitive function has also been accompanied with elevated hippocampal glycogen levels in normal rats^16^. In our very recent study, 4 weeks of moderate exercise is effective in improving the declining memory function (hippocampal) in type 2 diabetic rats, and this exercise-induced hippocampal-memory amelioration has been associated with hyper-glycogen levels in the hippocampus^23^. Indeed, recent studies have demonstrated that pharmacological or genetic inhibition of hippocampal glycogen metabolism impairs memory formation and compromises endurance capacity^19–22,24^. Furthermore, pharmacologically elevated brain glycogen levels in the brain protect neuronal activities under insulin-induced severe hypoglycemia^25^. Therefore, if GL increases brain glycogen levels as it does in muscle, GL is a possible strategy to enhance brain functions relating with memory and endurance performance.

To study the effects of GL on brain, determination of an appropriate GL condition for animals is needed. To date, Shinohara’s 1 week GL model, which is composed of an exhaustive exercise followed by a moderate exercise (10 min with a weight equal to 1–2% of body mass) and then rest with HCD, is useful as a reference because in their model hyper-glycogen storage has appeared in rat muscle after the GL^8^. However, their analysis is inadequate since the roles of the respective components of GL, namely EX (exhaustive exercise followed by moderate exercise and rest) and HCD, on glycogen storage is still unclear, and they did not address brain glycogen storage.

**Figure 1:**
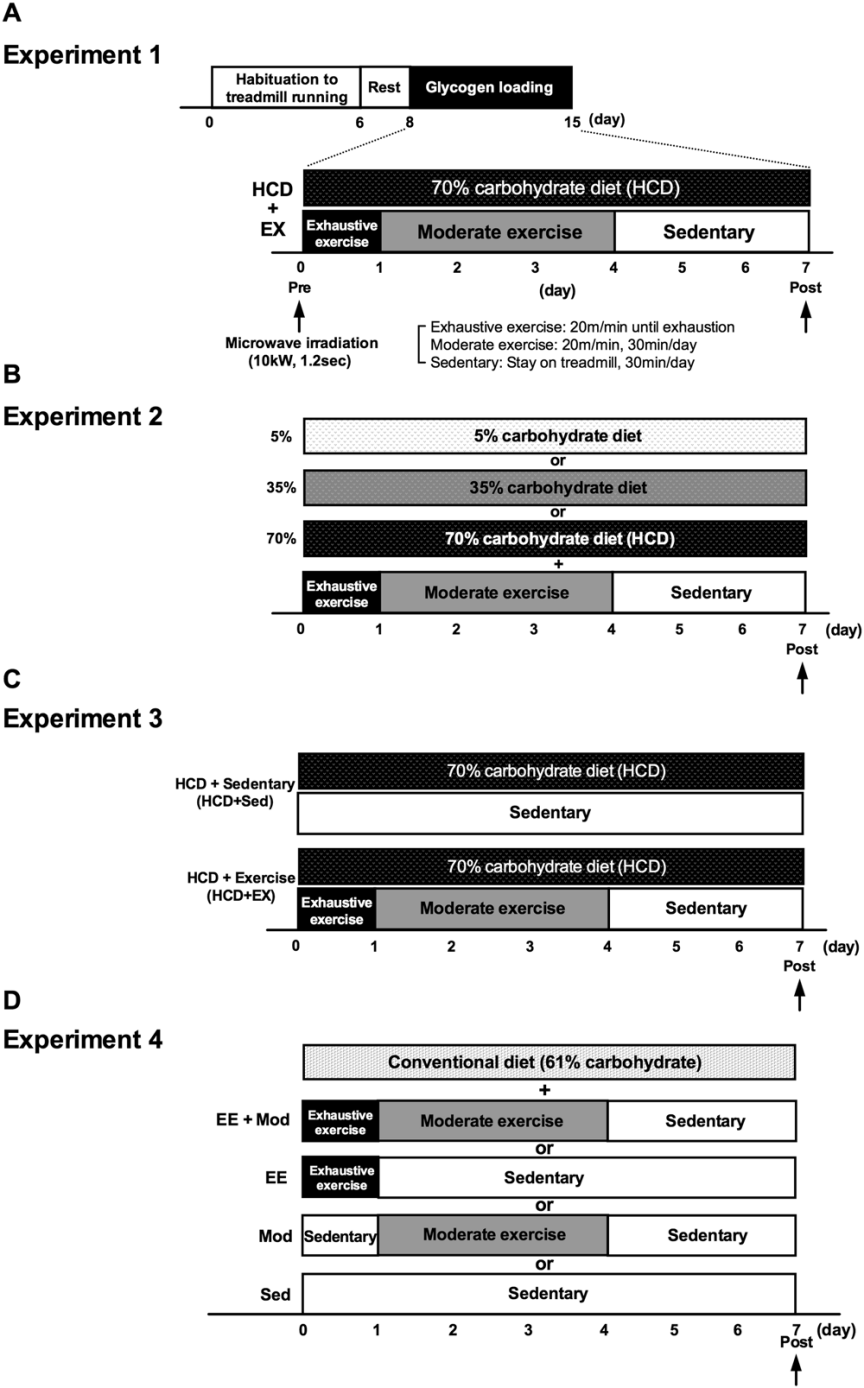
Experimental design. (**A**) One-week GL protocol for rat model (Experiment 1). Rats were divided into a Pre-GL and a Post-GL group, and underwent GL protocols with HCD (70% carbohydrates) and EX (day 1: exhaustive exercise (20 m/min until exhaustion), days 2–4: moderate exercise (20 m/min, 30 min/day), days 5–7: sedentary (rest on treadmill, 30 min/day)). Rats were sacrificed using high-power microwave irradiation (10 kW, 1.2 sec). (**B**) GL protocol to investigate the importance of the HCD component during GL (Experiment 2). Rats were divided into three diet groups each with different amount of carbohydrate (low: 5%, middle: 35%, and high: 70%) and fixed EX. Each group underwent GL protocol. (**C**) GL protocol to investigate the importance of the EX component during GL (Experiment 3). Rats were divided into two groups (HCD + EX and HCD + Sed), and underwent GL protocol. (**D**) GL with conventional-diet protocol to investigate the essential exercise condition during GL (Experiment 4). Rats were divided into four exercise groups (EE + Mod, a combination of exhaustive exercise and moderate exercise; EE, exhaustive exercise alone; Mod, moderate exercise alone; and Sed, sedentary), and underwent GL with a conventional diet (61% carbohydrates).

We thus performed four experiments as follows: First, we employed GL protocols, EX with HCD, in rat models and assessed whether glycogen levels increased after GL in various regions of the brains especially in the hippocampus, and in muscle (Experiment 1, Fig. 1A). Subsequently, we examined which GL component is dominant, EX or HCD, in inducing hyper-glycogen levels in the brain (HCD: Experiment 2, Fig. 1B; EX: Experiment 3, Fig. 1C, exercise conditions: Experiment 4, Fig. 1D). Through these analyses we intended to clarify whether GL or one of its two main components (EX and HCD) may have positive effects on brain glycogen storage.

## Results

### GL increases glycogen levels in both muscle and brain

**Figure 2:**
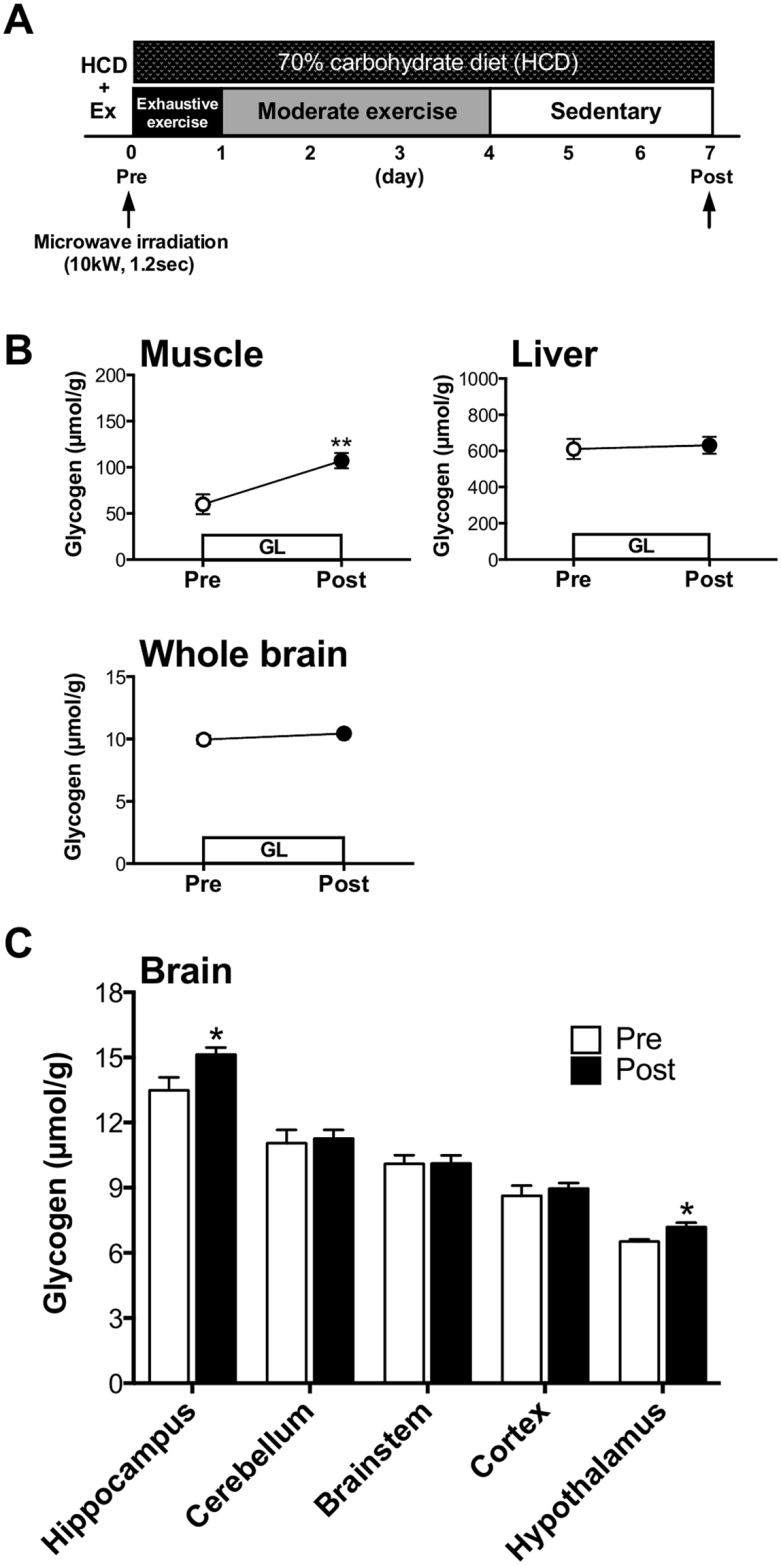
GL increased glycogen levels in muscle and the brains. (**A**) Experimental design. (**B**) Glycogen levels in the whole brain, muscle, and liver for pre- and post-GL. (**C**) Glycogen levels in the five brain loci (hippocampus, cerebellum, brainstem cortex and hypothalamus). Data are expressed as mean ± standard error (n = 6–8/group). *P < 0.05; **P < 0.01 versus pre-GL group (unpaired *t* test).

Rats underwent 1-week of GL, which consisted of several EX conditions and a HCD. Glycogen levels in muscle, liver and brain after GL were measured by using microwave irradiation (Fig. 2A). Muscle glycogen, but not liver glycogen, increased after GL (EX with HCD) (*P* < 0.01; Exp. 1, Fig. 2B) compared to pre-GL. Concomitantly, GL also led to increased brain glycogen levels, particularly in the hippocampus and hypothalamus (*P* < 0.05; Exp. 1, Fig. 2C). The rates of glycogen increase in muscle, the hippocampus and the hypothalamus were 79%, 12%, and 10%, respectively, implying that the GL model is effective for increasing brain glycogen, although the extent of the increase is much smaller than that of muscle.

### HCD during GL is not necessary for GL-induced hyper-brain glycogen levels in the brain

**Figure 3:**
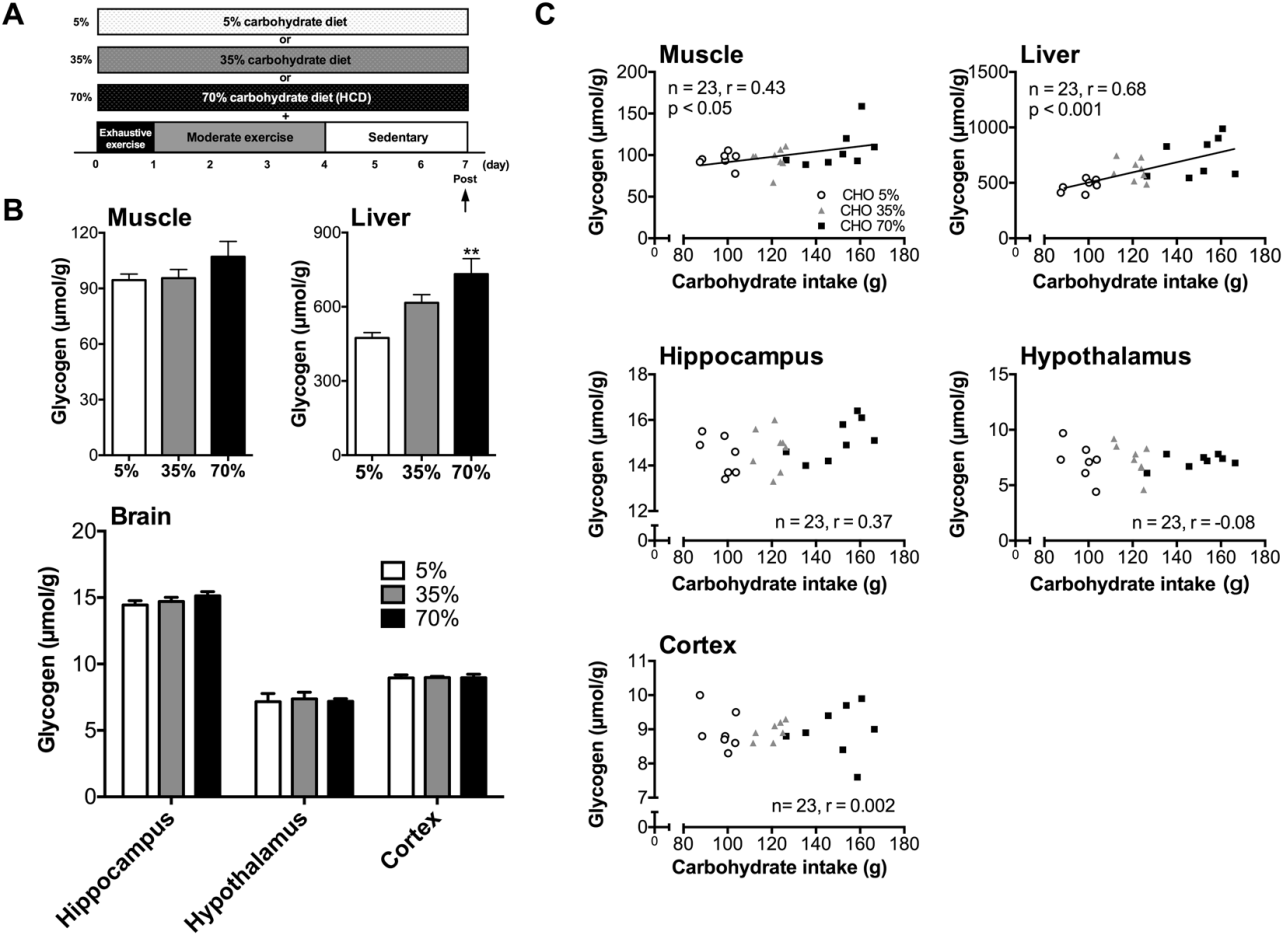
GL-induced hyper-glycogen in the muscle, but not in brain, is associated with carbohydrate intake. (**A**) Experimental design. (**B**) Glycogen levels in muscle, liver, and brain (hippocampus, hypothalamus, and cortex). Data are expressed as mean ± standard error (n = 7–8/group) **P < 0.01 versus 5% group (Dunnett’s *post hoc* test). (**C**) Correlation between carbohydrate intake and glycogen levels in muscle, liver, hippocampus, hypothalamus and cortex. Data are expressed as mean ± standard error (n = 7–8/group). Correlations are shown between the carbohydrate intake and glycogen levels. Lines in the scatter plots show significant correlation (by Pearson’s product-moment correlations test).

Rats were performed GL protocols with EX and different percentage of carbohydrate during GL (Fig. 3A). Three kinds of diets were adjusted same calorie per gram to examine whether HCD component in GL is necessary to induce hyper-hippocampal glycogen levels (Tables 1 and 2). During GL, only total carbohydrate intake increased with the increase of carbohydrate content in the diet (*P* < 0.001; Exp. 2, Table 3). Muscle glycogen levels did not differ significantly (Exp. 2, Fig. 3B) as the percentage of carbohydrates diet increased, but had a positive correlation with total carbohydrate intake (*r* = 0.43, *P* < 0.05; Exp. 2, Fig. 3C). In contrast, liver glycogen levels of the 70% carbohydrate diet group were 54% higher than those of the 5% carbohydrate diet group (Exp. 2, Fig. 3B). Furthermore, there was a positive correlation between carbohydrate intake and liver glycogen levels (*r* = 0.68, *P* < 0.001; Exp. 2, Fig. 3C). Regarding the relationship with fat intake and glycogen levels, there was significant negative correlation between fat intake and liver glycogen levels (*r* = −0.6, *P* < 0.01; Exp. 2, Fig. S2B), and a similar tendency was seen also in the muscle glycogen (*r* = −0.35, *P* = 0.09; Exp. 2, Fig. S2A). Of note, there was no significant change in brain glycogen levels in the carbohydrate-dose-dependent experiment without a significant correlation with carbohydrate intake (hippocampus: *r* = 0.37, *P* = 0.08, hypothalamus: *r* = −0.08, *P* = 0.71, cortex: *r* = 0.002, *P* = 0.99) (Exp. 2, Fig. 3B and C). Thus, muscle and brain glycogen levels may have similar responses to a gradual increase of carbohydrates in the diet during GL while their correlation with total carbohydrate intake was organ specific.

**Table 1:** Compositions of diets with different amounts of carbohydrates.

Ingredients/Diet	5%	35%	70%
Casein	23.1	23.1	23.1
DL-methionine	0.3	0.3	0.3
Corn starch	0.0	27.8	60.1
Sucrose	4.6	4.6	4.6
Corn oil	9.6	5.5	0.7
Lard	19.2	10.9	1.4
Cellulose powder	38.5	23.1	5.1
AIN-76 mineral mix	3.5	3.5	3.5
AIN-76 vitamin mix	1.0	1.0	1.0
Choline bitartrate	0.2	0.2	0.2
Total (%)	100.0	100.0	100.0

**Table 2:** Carbohydrate, fat, and protein ratios of three diets.

Diet	Nutrients	Content (g)	Calorie ratio (%)	Energy (kcal/100 g)	Total calorie (kcal/100 g)
5%	Carbohydrate	4.6	5.0	18.5	370.0
	Fat	28.8	70.0	259.0	
	Protein	23.1	25.0	92.5	
35%	Carbohydrate	32.4	35.0	129.5	370.0
	Fat	16.4	40.0	148.0	
	Protein	23.1	25.0	92.5	
70%	Carbohydrate	64.8	70.0	259.0	370.0
	Fat	2.1	5.0	18.5	
	Protein	23.1	25.0	92.5

**Table 3:** Baseline physiological variables after GL.

Parameter/Carbohydrate ratio in diet	5%	35%	70%
Body weight (g)	296.0 ± 3.2	298.0 ± 3.1	295.0 ± 4.4
Blood glucose (mM)	4.8 ± 0.1	4.5 ± 0.1	4.8 ± 0.1
Total food intake (g/6 days)	92.6 ± 5.9	90.7 ± 4.3	94.5 ± 6.5
Total calorie intake (kcal/100 g/6 days)	273.0 ± 20.0	268.5 ± 14.7	285.0 ± 24.0
Total carbohydrate intake (g/6 days)	3.6 ± 0.3	25.4 ± 1.4^***^	54.1 ± 4.5^***^

Data are expressed as mean ± standard error (n = 7–8/group). ***P < 0.001 versus 5%.

### Exercise during GL is required for GL-induced hyper-glycogen levels in muscle and brain

**Figure 4:**
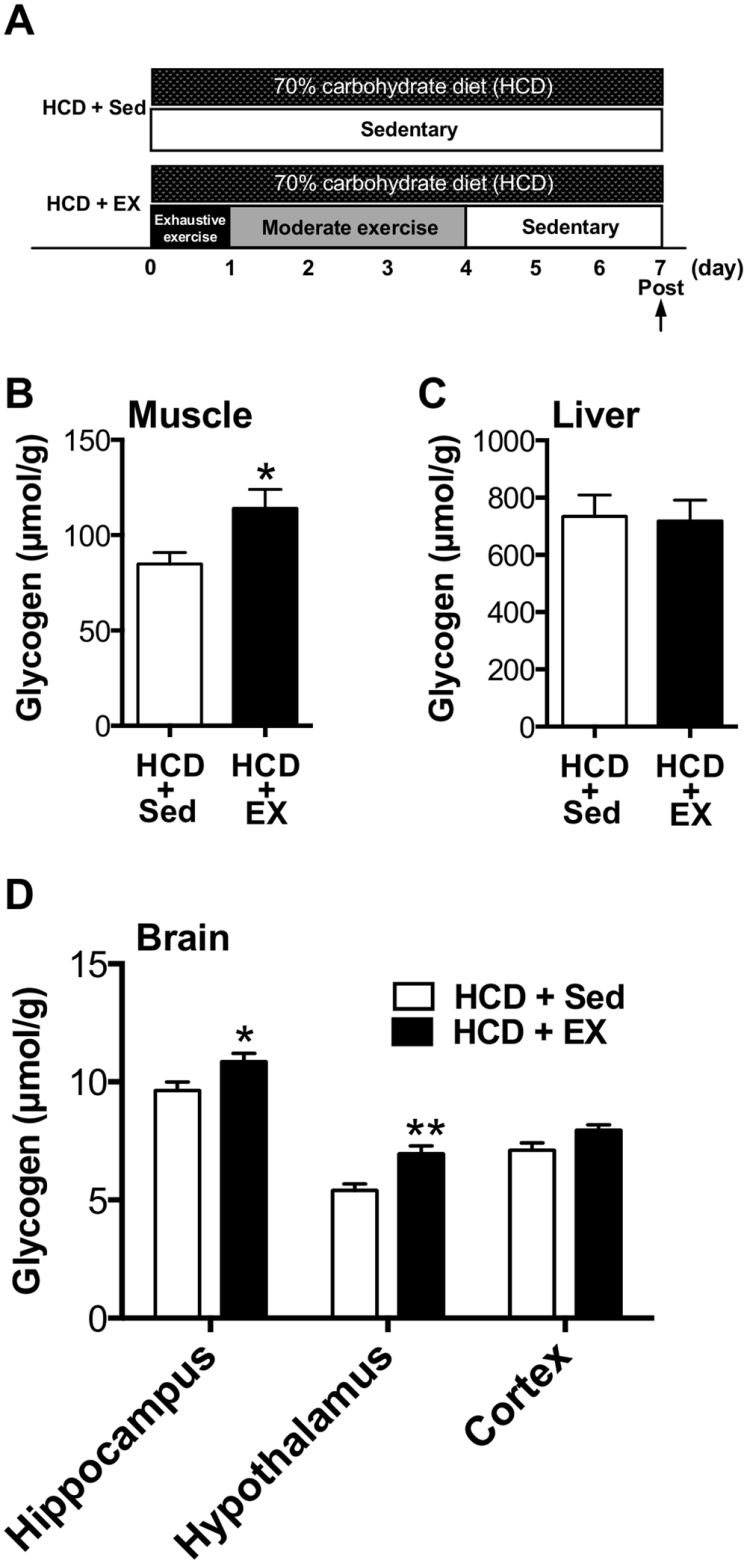
EX during GL is required for GL-induced hyper-glycogen in the muscle and the brain. (**A**) Experimental design. (**B**) Muscle glycogen. (**C**) Liver glycogen. (**D**) Brain glycogen levels in hippocampus, hypothalamus, and cortex. Data are expressed as mean ± standard error (n = 4–5/group) *P < 0.05; **P < 0.01 versus HCD ± Sed group (unpaired *t* test).

Since GL is composed of HCD and EX, next, we examined whether EX component in GL is necessary inducing hyper-hippocampal glycogen levels (Fig. 4A). Muscle glycogen levels increased with exercise (*P* < 0.05; Exp. 3, Fig. 4B). Brain glycogen levels in the hippocampus and hypothalamus, but not in the cortex, increased with EX (*P* < 0.05; Exp. 3, Fig. 4D). The rates of glycogen increase in muscle, the hippocampus and the hypothalamus were 34%, 13%, and 29%, respectively, implying that the GL, particularly the EX component, is crucial for increasing muscle and brain glycogen levels and, in particular, EX-induced brain glycogen increases are region specific.

### Exhaustive exercise is an essential for inducing hyper-glycogen levels in the hippocampus without a HCD

**Figure 5:**
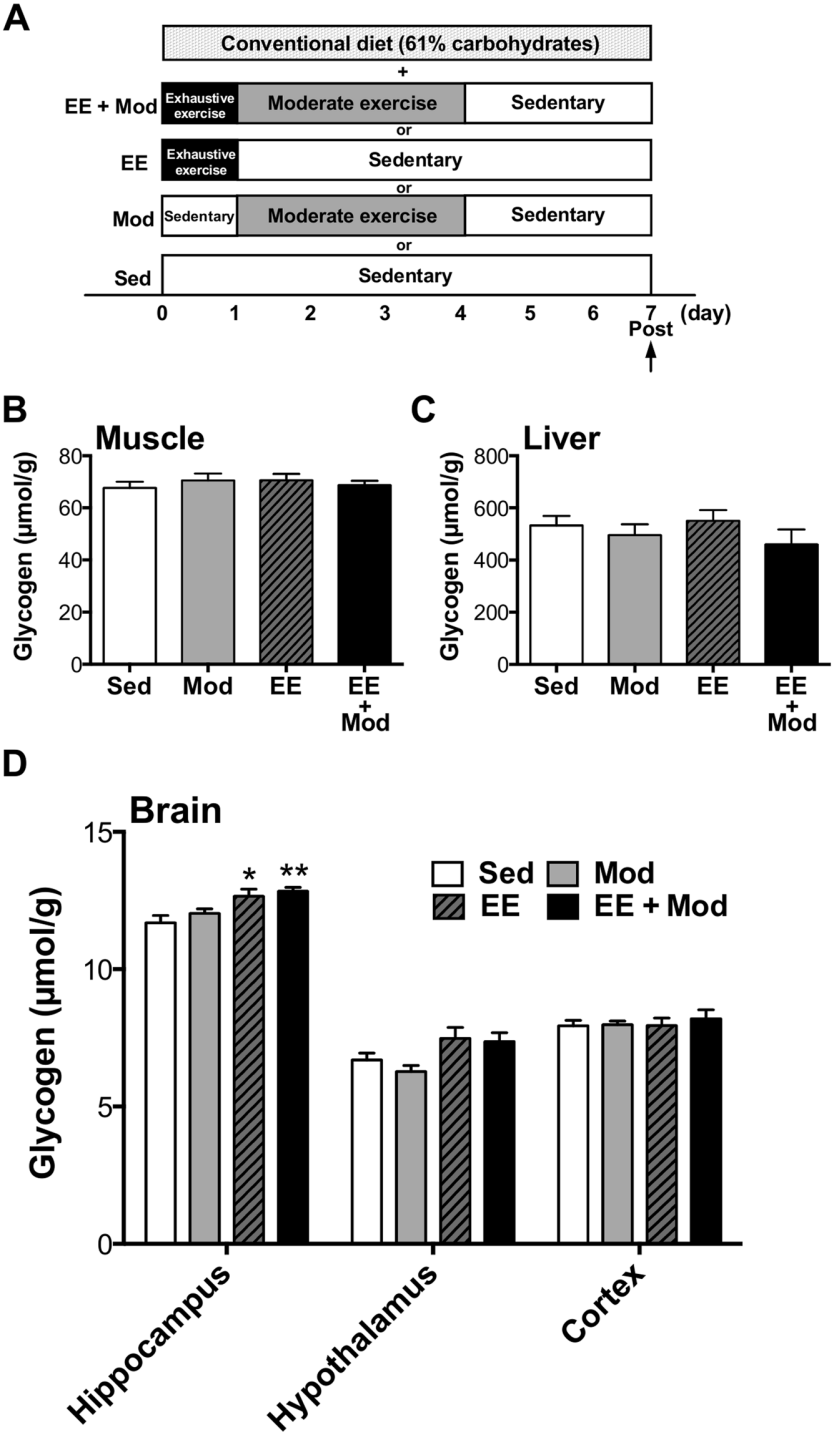
Exhaustive, but not moderate, exercise in GL icreasesd hippocampal glycogen with conventional diet. (**A**) Experimental design. (**B**) Muscle glycogen. (**C**) Liver glycogen. (**D**) Hippocampal, hypothalamic and cortical glycogen. Data are expressed as mean ± standard error (n = 5–10/group). *P < 0.05; **P < 0.01 versus sedentary group (Dunnett’s *post hoc* test).

Finally, we tried to determine which conditions of exercise (EE + Mod: a combination of exhaustive exercise and moderate exercise, EE: exhaustive exercise alone, Mod: moderate exercise alone, Sed: and sedentary) during GL protocol is essential for inducing hyper-hippocampal glycogen levels, thus, modified GL model, which consisted of several conditions of exercise and conventional diet (61% carbohydrate) was used in this experiment (Fig. 5A). Muscle and liver glycogen levels were unchanged in all exercise regimen groups irrespective of exercise conditions (Exp. 4, Fig. 5B,C). However, glycogen levels in the hippocampus, but not in the hypothalamus or cortex, increased with exhaustive exercise alone (8%) or with a combination of exhaustive and moderate exercise (10%) (*P* < 0.05; Exp. 4, Fig. 5D), implying that exhaustive exercise alone is sufficient to induce hyper-glycogen levels in the hippocampus with the possibility that this is a hippocampal-specific novel innovation of GL for the brain.

## Discussion

In the present study, we aimed to clarify whether GL or one or both of its two main components, EX (exhaustive exercise followed by moderate exercise and rest) and HCD (70% high carbohydrates diet), have positive effects on brain glycogen storage. We first tested the hypothesis that 1-week GL model of previous trials, EX with HCD^6,8^, woud have more impact on glycogen storage in some brain regions in a similar manner as in muscle, and, if so, we sought to better characterize the new GL effect of inducing hyper-glycogen storage in the brain in terms of the essential components of the GL using the GL protocol (see experimental protocol in Fig. 1). As a result, we have demonstrated that GL increases brain glycogen, at least in the hippocampus and hypothalamus with concomitant muscle glycogen accumulation. Further, the hyper-glycogen levels induced in the hippocampus by the GL are not dependent on carbohydrate intake during the GL, but are dependent on exhaustive exercise, a one of the EX components, implying the physiological significance of EX in enhancing brain glycogen synthesis.

One week after the GL, we found that brain glycogen levels had increased significantly in the hippocampus and hypothalamus as well as in muscle (pre-post muscle glycogen increase rate in ours vs. previous, 79% vs. 24%) (Exp. 1, Fig. 2B and C), which is consistent with previous studies in humans and rats^5–8^. These results showed that not only the validity of the current GL model for muscle glycogen storage, but also newly observed positive effects on glycogen storage in the brain, particularly in the hippocampus and hypothalamus (Exp. 1, Fig. 2C). Previous studies have shown that brain glycogen decreases with severe physiological conditions but it does not reduce after exposure to milder stimuli (e.g. fasting for 24 hours, sleep deprivation for over 12 hours, exhaustive exercise for 2 hours, etc.)^17,26,27^. In particular, Matsui *et al*.^17^ showed that 30 minutes of moderate exercise, which is the same condition with moderate exercise (day 2 to day 4) of GL, does not decrease brain glycogen levels. Thus, the combination of HCD and EX might contribute to the GL-induced hyper-glycogen levels in the hippocampus and hypothalamus as well as in the muscle. Furthermore, we examined the dynamics of muscle glycogen levels during GL (Fig. S1A) and confirmed increased glycogen at day 5–7 compared to pre-GL (*P* < 0.01) (Fig. S1). The GL-induced peak and timing of glycogen increase in the muscle are also consistent with previous studies^5–8^, indicating the physiological validity of our rat model mimicking human GL. These findings suggest the potential application of GL-induced hyper-hippocampal glycogen to enhance not only the endurance capacity but also the cognitive function of athletes and normal subjects.

Next, we examined which GL component is more dominant in inducing hyper-glycogen stores, the HCD or EX component, in Exp. 2 and 3. First, we examined the role of the HCD component in GL-induced hyper-brain glycogen storage in the hippocampus. In Exp. 2 (Fig. 3), food intake, total calorie intake during the GL were unchanged in the three groups with different amounts of carbohydrates in their diets (5%, 35%, and 70%). Furthermore, there was no significant difference between body weight and blood glucose among the three diet groups. Therefore, it is unlikely that insulin resistance occurred through the 5%-carbohydrate (high fat) diet. In these groups only the total carbohydrate intake increased, dependent on the percentage of carbohydrates in their respective diet (*P* < 0.001) (Table 3), and the results showed that the hyper-glycogen stores in all tissues of the three groups were not impacted (Fig. 3B). Furthermore, glycogen levels in peripheral tissues (liver and muscle) depended upon and had a positive correlation with the amount of carbohydrate intake, whereas brain glycogen levels did not, suggesting that hyper-glycogen levels in the brain (hippocampus and hypothalamus) occurred independently of carbohydrate intake (Fig. 3C). Regarding the liver, there was a negative correlation between fat intake and liver glycogen. However, the recovery of liver glycogen levels after exercise, which is the basis of GL, is strongly affected by carbohydrate intake^28^, furthermore, there is a strong positive correlation between carbohydrate intake and liver glycogen levels in this experiment 2 (Fig. 3C), suggesting the importance of carbohydrate intake increasing glycogen levels in liver.

As for a EX, we examined the effects of EX component in GL-induced hyper-hippocampal glycogen storage and found that EX with a HCD showed a significant hyper-hippocampal glycogen storage rather than Sed with HCD in both muscle and brain tissues (hippocampus and hypothalamus) (Fig. 4B and D) as well as pre-post changes in Exp. 1 (Fig. 2B). The present study provided a new hypothesis that EX is more dominant than HCD in terms of hyper- hippocampal glycogen stores. We further examined the effects of EX combined with a conventional diet (61% carbohydrates) and found that exhaustive exercise (EE) with and without moderate exercise (Mod) (EE + Mod group: exhaustive exercise followed by moderate exercise and rest, EE group: exhaustive exercise followed by rest) resulted in similar hyper-glycogen stores only in the hippocampus (Exp. 4, Fig. 5D). The Mod (moderate exercise followed by sedentary) and Sed (sedentary) conditions produced no effects on hyper-glycogen storage in the hippocampus (Exp. 4, Fig. 5D). Thus, the implementation of exhaustive exercise itself is a crucial factor in long-term (one week) hyper-glycogen storage in the hippocampus. In our previous study, supercompensation occurs rapidly in brain glycogen^16^: within 6 hours after exhaustive exercise; the rates of supercompensation peak were 29–60% in the brain (whole brain: 46%, cortex: 60%, hippocampus: 33%, hypothalamus: 29%, etc.), and 46% in the plantaris muscle at 24 h after exhaustive exercise. Additionally, significant increases remained at 24 h after exercise in the hippocampus and cortex^16^. While the mechanism remains unknown, the hyper- glycogen stores in the hippocampus over one week might be in part due to the first exhaustive exercise-induced supercompensation. Finally, EX with HCD showed a significant hyper-glycogen storage rather than Sed with HCD in both muscle and brain tissues (hippocampus and hypothalamus) (Fig. 4B and D) as well as pre-post changes in Exp.1 (Fig. 2B). On the other hand, a conventional diet did not lead to increased muscle glycogen (Exp. 4, Fig. 5B), but a HCD did (Exp. 3, Fig. 4B). These results further suggest the importance of a HCD, not EX alone, in inducing hyper-glycogen levels in muscle, supporting the results that GL induces increased hyper-glycogen levels in muscle in a manner dependent on carbohydrate intake.

We found that 1 week of GL with HCD resulted in hyper-glycogen stores only in the hippocampus (Fig. S1), which were kept at the same levels during last three days of GL, probably due to an altered set-point of brain glycogen synthesis and usage. Although the role and underlying mechanisms of hyper-hippocampal glycogen stores remains unclear, noradrenaline might be involved. Noradrenergic neuron are activated during intense/prolonged acute exercise^17,29–32^, and its noradrenergic metabolism is associated with an exercise-induced brain glycogen decrease^17^. Meanwhile, in cultured astrocytes, noradrenaline injection not only stimulates glycogenolysis within 30–60 minutes, but also stimulates its supercompensation *via* the expression of protein targeting to glycogen (PTG), an activator for glycogen synthase, 4–24 hours after injection^33,34^. Such the intriguing noradrenergic metabolic dynamics of astrocytic glycogen is consistent with an acute exercise effect^17^, suggesting a possible role of noradrenaline in the post-exercise hippocampal glycogen supercompensation contributing to the long-term hyper-hippocampal glycogen stores in this study.

What is the role of the hippocampal hyper-glycogen stores? Current studies show that hippocampal glycogen contributes to memory formation *via* lactate production and transportation^21,22^, and this is also the case in exercise endurance^19^. Exploring, with the use of behavioral study^21,22^, immunohistochemistry for measuring brain glycogen^35^, and LTP assessment^22^, whether or not such hyper-glycogen stores enhance memory should be the next step in this assessment.

Our findings provide the first evidence that GL, a combination with HCD and EX, increases brain glycogen, at least in the hippocampus and hypothalamus, with concomitant muscle glycogen deposition. Further, the increase in GL-induced hyper-glycogen levels in the hippocampus does not depend on carbohydrate intake, but does depend on exhaustive exercise, one component of EX (exhaustive exercise followed by moderate exercise and rest), implying the physiological significance of exercise in enhancing brain glycogen synthesis. This new perspective on exercise and brain glycogen will ultimately lead to novel sports/nutrition conditioning for memory function and exercise endurance.

## Methods

### Materials

All chemicals, including amyloglucosidase, hexokinase, NADP+-dependent glucose-6-phosphate dehydrogenase, NADP^+^, ATP, EDTA, MgSO_4_, glucose, glucose-6-phosphate, KOH, imidazole, perchloric acid, and Tris-HCl are from Sigma (St Louis, MO, USA) and Nacalai tesque (Kyoto, Japan).

### Animals

Adult male Wistar rats (250–270 g; SLC Inc., Shizuoka, Japan) were housed individually, cared for in an animal facility and fed a Conventional diet (Oriental Yeast Co., Ltd, Ibaraki, Japan) with free access to water from the first week of acclimatization to habituation to treadmill running. The composition of the Conventional diet was 26% protein, 13% fat, and 61% carbohydrates. The room temperature was maintained between 22 and 24 °C under a 12:12 hours light/dark cycle (light on 7:00–19:00). All experimental protocols were approved by the Institutional Animal Care and Use Committee of the University of Tsukuba, and all procedures and methods were performed in accordance with the relevant guidelines laid down by animal ethics committee (Animal ethical approval number; 15–055). Every effort was made to minimize the number of animals used as well as any pain and discomfort.

### Habituation to treadmill running

Rats were habituated to running on a treadmill (SN-460, Shinano, Tokyo, Japan) for a total of 5 sessions over 6 days after a 1-week acclimatization period. The running duration was 30 min/day, and the running speed was gradually increased from 5 to 25 m/min^16,17,19,36^.

### Experimental procedures

#### Experiment 1

The experimental design of Experiment 1 is shown in Fig. 1A and Fig. 2A. Two days after the habituation period, rats underwent 1-week of GL, which consisted of several EX conditions and a HCD. The GL protocol in the present study was a modified version of 1-week muscle GL protocol that has been described previously^8^. Rats were fed a 70% carbohydrate (HCD) powder diet (Oriental Yeast Co., Ltd, Ibaraki, Japan) during the GL period. The composition of the HCD was 25% protein, 5% fat, and 70% carbohydrates. First, rats were divided randomly into a pre-GL and a post-GL group. As for EX protocol, on day 1 of the GL period, rats initially performed exhaustive exercise (EE): treadmill running at moderate intensity (20 m/min) until exhaustion, which has been determined as 50–70% VO_2_max for rats. Exhaustion was considered to have occurred when the rat was unable to keep pace with the treadmill, lay flat, and stayed on the grid positioned at the back of the treadmill for a period of 30 seconds despite being gently pushed with sticks or breathed on^16,17,19,37^. From day 2 to 4, rats performed additional moderate-intensity exercise (Mod) (20 m/min, 30 min/day), and then rats were allowed to rest (Sed) on the treadmill (0 m/min, 30 min) from day 5 to 7. Rats were sacrificed using microwave irradiation on day 0 (Pre) and day 7 (Post) in GL. Following microwave irradiation, five brain loci (the cortex, hippocampus, hypothalamus, cerebellum and brainstem) were collected using a method modified from Hirano *et al*.^38^. Skeletal muscle and liver were also collected.

#### Experiment 2

The experimental design of Experiment 2 is shown in Figs 1B and 3A. Another series of rats divided into two groups (5%, 35%, and 70%) and underwent GL protocols with various percentages of carbohydrates (5%, 35%, and 70% (HCD)) in their diets and EX protocol as for the GL protocol mentioned above. An experimental approach using calorie-controlled diets by adjusting the amount of cellulose powder is standard in the field of nutrition^11,39,40^, thus, the calories of the three diet groups were unified (Tables 1 and 2). Finally, rats were sacrificed using microwave irradiation on day 7 in GL. Following microwave irradiation the hippocampus and hypothalamus were collected along with the cortex as a negative control for brain regions relating to the cognitive function. These same brain regions were also collected in subsequent experiments as well. Skeletal muscle and liver were also collected.

#### Experiment 3

The experimental design of Experiment 3 is shown in Figs 1C and 4A. Another series of rats divided into two groups (HCD + Sed, HCD + EX) and underwent GL protocols with HCD and Sedentary or EX protocol as for the modified GL protocol mentioned above. Finally, rats were sacrificed using microwave irradiation on day 7 in GL.

#### Experiment 4

The experimental design of Experiment 2 is shown in Figs 1D and 5A. Another series of rats were divided into four groups (EE + Mod: a combination of exhaustive exercise and moderate exercise, EE: exhaustive exercise alone, Mod: moderate exercise alone, Sed: sedentary alone) and underwent GL with various exercise conditions and a conventional diet (61% of carbohydrates) for 1 week. Finally, rats were sacrificed using microwave irradiation on day 7 in GL.

### Tissue preparation

Rats were anesthetized with isoflurane (a mixture of 30% vol/vol isoflurane in propylene glycol; Dainippon Sumitomo Pharma Co., Ltd., Osaka, Japan) in a bell jar and sacrificed using high-power microwave irradiation (NJE-2603, New Japan Radio Co., Ltd., Tokyo, Japan; 10-kW, 1.2 sec). Following the microwave irradiation, 5 brain loci (hippocampus, cortex, hypothalamus, cerebellum and brainstem) were collected. Muscle (plantaris and/or soleus), liver, and blood samples were also collected. All tissue samples were stored at −80 °C for subsequent biochemical analysis.

### Blood glucose, lactate assays

Whole-blood glucose and lactate levels in the whole blood were measured using an automated glucose-lactate analyzer (2300 Stat Plus, Yellow Springs Instruments, USA).

### Glycogen assay

The methods of glycogen and glucose measurement were consistent with the method of our previous studies^16,17,19^. Tissues were homogenized (Polytron, Kinematica, Kriens-Luzern, Switzerland; 4,100 *g*, 30 seconds, 3 times) in ice-cold 6% perchloric acid (PCA) containing 1 mM EDTA. For tissue glycogen content measurements, glycogen in 100 µl of homogenate was hydrolyzed to glucose by incubating for 3 hours at 37 °C with 1 ml of 0.2 M sodium acetate, 20 µl of 1.0 M KHCO_3_, and 20 U/ml of amyloglucosidase. To stop the subsequent enzymatic reaction, 0.5 ml of PCA was added. After centrifugation (14,000 *g*, 10 minutes, 4 °C), and supernatants were neutralized with a KOH solution, consisting of 3 M KOH, 0.3 M imidazole and 0.4 M KCl. The supernatants were then centrifuged (16,000 *g*, 10 minutes, 4 °C) and measured for glucose content. To measure endogenous glucose levels, non-hydrolyzed samples were obtained by centrifuging homogenates (14,000 *g*, 10 minutes, 4 °C) and the pH of the supernatants was controlled to a final pH of 6–8 with KOH solution. Neutralized samples were mixed thoroughly, centrifuged (16,000 *g*, 10 minutes 4 °C), and measured for endogenous glucose levels. The glucose content measurement was performed in 96-well plates using a coupled enzyme assay method. A total of 200 µl of a reaction solution including 50 mM Tris-HCl (pH 8.1), 0.5 mM ATP, 0.5 mM NADP^+^, 5 mM MgSO_4_, and 0.1 U/ml glucose-6-phosphate dehydrogenase was added to each well. Then, the plate was placed in the fluorescence plate reader (Arvo, Perkin Elmer, Groningen, Netherlands) and shaken, and measurements of the resultant NADPH were taken at 350 nm excitation and 450 nm emission. The plates were shaken after the addition of hexokinase (0.3U) to each well, and measurements were taken after a 30-min incubation period. Tissue glycogen levels, indicted as glucose units, were calculated by subtracting the final micromolar concentration of glucose per gram of wet weight of the non-hydrolyzed tissue sample from the final micromolar concentration of glucose per gram of wet weight of the hydrolyzed sample.

### Statistical analyses

Data are expressed as mean ± standard error (SEM) and were analyzed using Prism 5 (MDF Co., Ltd, Tokyo, Japan). Comparisons of two groups were performed using Student’s *t* test for unpaired data. Group comparisons were performed using a one-way ANOVA with Dunnett’s *post hoc* tests. Correlations were calculated using Pearson’s product-moment correlations. Statistical significance is P values < 0.05.

## Electronic supplementary material

Supplementary information
