# Supplementary material for: Hyper-hippocampal glycogen induced by glycogen loading with exhaustive exercise
Source: Sci Rep. 2018 Jan 19;8:1285. doi: 10.1038/s41598-018-19445-4 (PMC5775355; doi:10.1038/s41598-018-19445-4)
Supplement: Supplementary file 1 — Supplementary information [file 41598_2018_19445_MOESM1_ESM.doc]

**Hyper-hippocampal glycogen induced by glycogen loading with exhaustive exercise**

**Mariko Soya^1^, Takashi Matsui^1,3^, Takeru Shima^1^, Subrina Jesmin^1^,**

**Naomi Omi^2,4^ & Hideaki Soya^1,3^**

*^1^Laboratory of Exercise Biochemistry and Neuroendocrinology; ^2^Laboratory of Exercise Nutrition, Faculty of Health and Sport Sciences,*

*^3^Department of Sport Neuroscience; ^4^Department of Body,*

*Advanced Research Initiative for Human High Performance (ARIHHP),*

*Faculty of Health and Sport Sciences,*

*University of Tsukuba, Tsukuba 305-8574, Ibaraki, Japan*

**Corresponding author:**

**Hideaki Soya, Ph.D.**

Professor, Laboratory of Exercise Biochemistry and Neuroendocrinology

Director, Advanced Research Initiative for Human High Performance (ARIHHP)

University of Tsukuba Faculty of Health & Sport Sciences

Address: 1-1-1 Tennoudai, Tsukuba, Ibaraki 305-8574, Japan

E-mail: [soya.hideaki.gt@u.tsukuba.ac.jp](mailto:soya.hideaki.gt@u.tsukuba.ac.jp)

Tel/Fax: +81-29-853-2620

**Figure S1**

**Figure S1 Hyper-glycogen levels in the muscle and hippocampus is sustained in GL period.** **(A)** Experimental design. **(B)** Muscle glycogen. **(C)** Hippocampal glycogen. **(D)** Hypothalamic glycogen. **(E)** Cortical glycogen. Data are expressed as mean ± standard error (n = 7-8/group). **P* < 0.05; ***P* < 0.01; ****P* < 0.001 versus pre-GL group (Dunnett’s *post hoc* test).

**Figure S2**

**Figure S2 Correlation between fat intake and glycogen levels in the muscle, liver, and brain.** **(A)** Muscle. **(B)** Liver. **(C)** Hippocampus. **(D)** Hypothalamus. **(E)** Cortex. Data are expressed as mean ± standard error (n = 7-8/group). Correlations are shown between the fat intake and glycogen levels. Lines in the scatter plots show significant correlation (by Pearson’s product-moment correlation test).
